# Transcriptome Sequencing and Analysis of *Trichoderma polysporum* Infection in *Avena fatua* L. Leaves before and after Infection

**DOI:** 10.3390/jof10050346

**Published:** 2024-05-13

**Authors:** Haixia Zhu, Yushan He

**Affiliations:** 1 Academy of Agriculture and Forestry Sciences, Qinghai University, Xining 810016, China; yushanhe312@163.com; 2 State Key Laboratory of Plateau Ecology and Agriculture, Qinghai University, Xining 810016, China; 3 Key Laboratory of Agricultural Integrated Pest Management of Qinghai Province, Xining 810016, China

**Keywords:** transcriptome sequencing, *T. polysporum* HZ-31, *Avena fatua* L., key genes

## Abstract

Biological control is a scientific management method used in modern agricultural production, and microbially derived biopesticides are one effective method with which to control weeds in agricultural fields. In order to determine the key genes for weed control by *Trichoderma polysporum*, transcriptome sequencing was carried out by high-throughput sequencing technology, and the strains of *T. polysporum* HZ-31 infesting *Avena fatua* L. at 24, 48, and 72 h were used as the experimental group, with 0 h as the control group. A total of 690,713,176 clean reads were obtained, and the sequencing results for each experimental group and the control group (0 h) were analyzed. In total, 3464 differentially expressed genes were found after 24 h of infection with the pathogen, including 1283 down-regulated genes and 2181 up-regulated genes. After 48 h of infection, the number of differentially expressed genes was 3885, of which 2242 were up-regulated and 1643 were down-regulated. The number of differentially expressed genes after 72 h of infection was the highest among all the groups, with 4594 differentially expressed genes, of which 2648 were up-regulated and 1946 were down-regulated. The up-regulated genes were analyzed by GO and KEGG, and the results showed that the up-regulated differentially expressed genes were mainly enriched in the biosynthesis of phenylalanine, tyrosine, and tryptophan; the degradation of aromatic compounds; methane metabolism; and other pathways. Among them, the *PHA2*, *GDH*, *ADH2,* and *AROF* genes were significantly enriched in the above-mentioned pathways, so they were hypothesized to play an important role in the synthesis of the herbicidally active substances of *T. polysporum* HZ-31. The results of this study can provide a theoretical basis for further studies on the pathogenicity of *T. polysporum* to *A. fatua* L., and accelerate the development and utilization of new and efficient bioherbicides.

## 1. Introduction

*Avena fatua* L. is considered one of the most serious weeds in the world [1], and it poses a serious economic threat to crop yields due to its unique seed traits, including high competitiveness, staggered germination, chemosensory potential, and the ability to persist in the soil seed bank [2]. *A. fatua* L. is the second-most-important grass weed in Australia, costing cereal growers more than AUD 10,000 per year in lost income [3]. In China, 60% of the wheat-sown area in Qinghai Province (about 8.7 million hectares of farmland) is damaged by *A. fatua* L. [4]. However, the use of chemical herbicides to control it faces numerous obstacles, the most difficult of which is the development of resistance, with *A. fatua* L. ranking second among the 10 weeds most likely to develop resistance. The selection of the wrong type of herbicide, prolonged use of a single type of herbicide, application of an insufficient dose, late application, and the use of generics can increase the risk of selecting resistant biotypes [5]. Therefore, there is an urgent need for new, safe, non-polluting, and efficient bioherbicides to deal with *A. fatua* L. control and resistance development. Microorganisms and their metabolites have herbicidal activity. Microbial herbicides developed using microbial metabolites, especially plant pathogenic toxins, usually have the characteristics of safety, providing environmental protection, high efficiency, and multiple target sites, which can achieve green and efficient control and complicate the development of weed resistance.

Its rapid growth and ability to metabolize a wide range of substrates make *Trichoderma* a major component of the soil flora in diverse ecosystems, such as agricultural fields, pastures, grasslands, forests, saline wetlands, deserts, and polar regions [6]. It has an effective antagonistic mechanism with which to inhibit the growth of plant pathogenic fungi, weeds, or nematodes, and is a source of biological control agents in agriculture. *Trichoderma polysporum* is a biocontrol fungus of the *Trichoderma* with anti-stress, antibacterial and anti-grass activities, and studies have shown that it possesses biocontrol activity against a variety of fungi, such as *Armilaria gallica* [7], *Pseudogymnoascus destructans* [8], *Alternaria panax* [9], and *Ceratocystis paradoxa* [10]. Previous studies in our laboratory have found that *T.polysporum* can also be highly effective for the inhibition of weeds, such as *A.fatua* L., *Chenopodium album* L., and *Polygonum lapathifolium*, and has the potential to be developed as a biological control agent for weeds [11]. Because *T.polysporum* can also produce secondary metabolites, such as cyclosporin [12], xylomycin [13], and anthraquinones [14], the secondary metabolites have been extracted and identified in our laboratory. It has been found that it can produce various active substances, such as 1, 8-propanediol-o-xylene and 2, 3-dihydroxypropyl propionate, which have an inhibitory effect on weeds [15]. Therefore, the above studies have indicated that *T.polysporum* is a fungal resource of high value to be utilized in the biological control of weeds.

Although *T. polysporum* HZ-31 has the potential to be exploited as a biocontrol agent, further research is needed to improve the efficiency of its biocontrol and to discover new genes and active secondary substances. Transcriptomics, the global analysis of gene expression at the RNA level, is a key element of functional genomics that will greatly contribute to our understanding of gene function in the post-genomic era [16]. Therefore, in this study, transcriptome sequencing was performed at different time points after the inoculation of *A. fatua* L. with *T. polysporum* HZ-31, and the sequencing data were comparatively analyzed to reveal the key pathways and genes involved in the infection of *A. fatua* L. by *T. polysporum* HZ-31. The results are expected to provide candidate resources for future research on key gene functions.

## 2. Materials and Methods

### 2.1. Test Strains

*T. polysporum* strain HZ-31 was isolated from the stem base of a diseased *Cirsium arvense var. setosum* by the Key Laboratory of Comprehensive Management of Agricultural Pests in Qinghai Province, and it was identified and preserved.

### 2.2. Infection of A. fatua L. with T. polysporum HZ-31 and Culture Treatments

A small amount of the mycelium of *T. polysporum* strain HZ-31, which was maintained in the Key Laboratory of Comprehensive Management of Agricultural Pests in Qinghai Province, was placed in the center of PDA medium and cultured in an incubator at 25 °C for 4–5 days. The cultured strain was then inoculated into new PDA medium by punching out the cake with a 5 mm circular punch, and then used for the subsequent experiments when it had grown for 5 days. The *A. fatua* L. leaves were washed three times in sterile water and placed in a petri dish with filter paper. Moist cotton wool was used to cover the breaks in the *A. fatua* L. leaves, and sterile water was added to the petri dish until the filter paper was slightly wet for the subsequent experiments. The edges of the cultured *T. polysporum* HZ-31 strains were punched, and the fungus cakes were randomly picked and inoculated on the surface of the *A. fatua* L. leaves, such that the side of the cakes with mycelium was guaranteed to be in contact with the *A. fatua* L. leaves, and they were placed in an incubator at 25 °C and sampled at 24 h, 48 h, and 72 h. When sampling, the cultured cakes were placed into 2 mL centrifugal tubes after removing the residues of the medium and *A. fatua* L. leaves as much as possible. Ten pieces were extracted from each centrifugal tube and clearly labeled, and the uninoculated cakes were used as the control. Each type of control and treatment were repeated three times, and then frozen in liquid nitrogen and stored at −80 °C.

### 2.3. RNA Extraction and Quality Testing of Samples

The total RNA was extracted using the TRNzol Universal Reagent extraction kit according to the extraction instructions. Qubit2.0 was used to measure the RNA concentration, and agarose gel was used to detect RNA integrity and genomic contamination.

### 2.4. Library Construction and Sequencing

The transcriptome library construction, sequencing, and assembly of the extracted samples were completed by Sangon Bioengineering (Shanghai) Co., Shanghai, China.

### 2.5. Analysis of Transcriptome Sequencing Information

#### 2.5.1. Data Quality Control

The raw data obtained from sequencing contains sequences with junctions and low quality. To ensure the quality of the information analysis, the raw data must be filtered to obtain the clean data. Data processing using Trimmomatic includes the removal of sequences with N bases; the removal of splice sequences in the reads; the removal of low-quality bases (Q-value < 20) starting from the 3′ to 5′ direction of the reads; the removal of low-quality bases (Q-value < 20) starting from the 5′ to 3′ direction to start removing the low-quality bases (Q-value < 20); removing bases with quality values below 20 in the tails of the reads using the sliding window method (window size of 5 bp); and removing the reads themselves and their paired reads, for lengths of reads less than 35 nt, to obtain the clean data.

#### 2.5.2. Reference Sequence Comparison Analysis

The clean data obtained from sequencing were compared with the reference genome of *T. polysporum* HZ-31 PRJNA941260 (https://www.ncbi.nlm.nih.gov/datasets/genome/?bioproject=PRJNA941260, accessed on 16 June 2023) using HISAT2 2.1.0, and the results of the comparison were statistically analyzed by RSeQC2.6.1.

#### 2.5.3. Expression Statistics

The gene expression levels were estimated by counting the sequenced sequences (reads) localized to the genomic regions or exonic regions of the genes. *TPM* (Transcripts Per Million) is a measure of the proportion of a particular transcript in a pool of RNAs. *TPM* accounts for both the sequencing depth and the length of the gene as well as the effect of the sample on the counts of the reads, and it is a commonly used method for estimating gene expression levels. The *TPM* formula is as follows:

$${TPM}_{i}=\frac{X_{i}}{L_{i}}*\frac{1}{\sum_{j}\frac{X_{j}}{L_{j}}}*10^{6}$$

$${X_{i}=total\;exon\;fragment/reads\;L}_{i}=\frac{exon\;length}{KB}$$

#### 2.5.4. Differential Expression Analysis

In order to screen the differential genes in the treatment and control groups of *A. fatua* L. infected with *T. polysporum* HZ-31, DESeq 1.26.0 was used to analyze the differential expression of the genes in each sample, and the screening conditions were as follows: DEGs with a q-value < 0.05 and a multiplicity of differences |fold change| > 2. The genes meeting these two criteria were considered to be significantly differentially expressed.

#### 2.5.5. Differential Gene Enrichment Analysis and Functional Annotation

GO (Gene Ontology) and KEGG (Kyoto Encyclopedia of Genes and Genomes) functional enrichment analyses were performed using clusterProfiler 3.0.5. GO enrichment was performed using topGO 2.24.0 alone because GO is a directed acyclic structure. In general, when the corrected *p*-value (Q-value) was < 0.05, the function was considered to be significantly enriched.

#### 2.5.6. Validation of Transcriptome Results

The samples used for qRT-PCR were from the same batch as the transcriptome sequencing. The cDNA first-strand synthesis was performed using a FastKing gDNA Dispelling RT SuperMix kit from Tengen (Beijing, China). Based on the analysis of the *T. polysporum* HZ-31 transcriptome sequencing results, 20 genes related to the biosynthesis of phenylalanine, tyrosine, and tryptophan; methane metabolism; the degradation of aromatic compounds; and the phenylalanine metabolism pathway were selected from the batch, and primers were designed and subjected to qRT-PCR experiments. The internal reference for qRT-PCR is Actin, a commonly used internal reference gene in fungi. The qRT-PCR reaction system was as follows: 2 × SuperReal Color PreMix, 25 μL; PCR Forward Primer (10 μM), 1 μL; PCR Reverse Primer (10 μM), 1 μL; cDNA, 1 μL; 50 × ROX Reference Dye, 0.5 μL; ddH_2_O, 21.5 μL. The reaction was carried out in a qRT-PCR instrument using the qRT-PCR reaction program: predenaturation at 95 °C for 15 min, 1×; denatured at 95 °C for 10 s; and annealed at 60 °C for 30 s, 40×. The primers are shown in Table 1.

## 3. Results and Analysis

### 3.1. Transcriptome Data Assembly Results

In order to screen the genes related to the prevention of infection of *A. fatua* L. by *T. polysporum* HZ-31, samples of the mycelium of *T. polysporum* HZ-31 were collected at 0, 24, 48, and 72 h of infestation of *A. fatua* L. for transcriptome sequencing, and quality control was carried out on the sample data after obtaining the raw data. As shown in Table 2, an average of 59,053,547 raw reads were measured, and the samples were filtered for the raw data to obtain an average of 57,559,431-retained clean reads. All the samples with Q20 above 98% (greater than 95%) and Q30 above 95% (greater than 80%) indicated high data quality, and the GC content was above 50%, which proved that the sequencing was not obviously biased. Therefore, the data quality was satisfactory.

### 3.2. Reference T. polysporum Genome Comparison Results

Using the *T. polysporum* genome measured in this article as the reference genome, the comparison results are shown in Table 3. The total reads are the sum of read1 and read2, which is known as the clean reads. The reads on the genome compared to the clean reads are more than 95%, so more than 95% of the reads in a unique position are compared, and less than 1% of the reads in multiple positions are compared. Both read1 and read2 are compared to the genome, with more than 47% and more than 92% respectively of the reads simultaneously compared to the genome at both ends.

### 3.3. Expression Analysis

**Figure 1:**
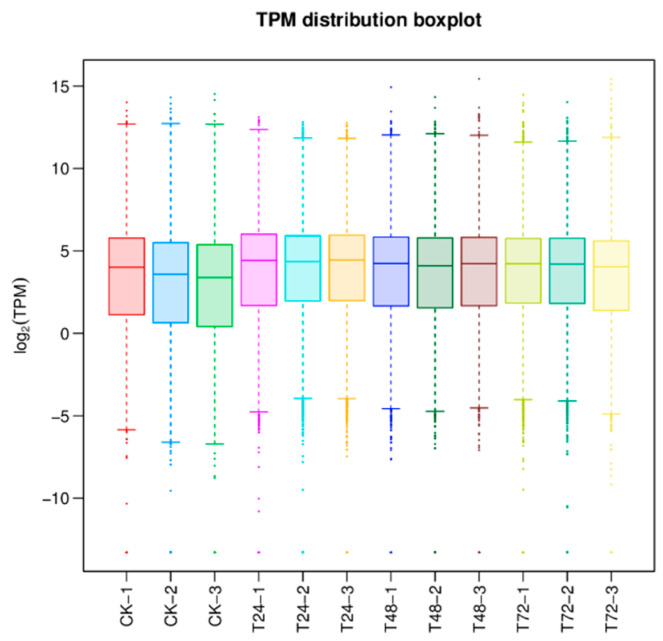
Results of gene expression analysis. The horizontal axis is the sample name and the vertical axis is the log(*TPM*) value. The box plot for each region is for five statistics (top to bottom for maximum, upper quartile, median, lower quartile, and minimum, respectively). Different colors represent different samples.

As shown in Figure 1, the highest, lowest, and median log2(*TPM*) values of gene expression in the different samples can be known according to the box line plot of gene expression. The results show that the discrete degree of gene expression of *T. polysporum* HZ-31 is more concentrated, the degree of difference is higher in the treatment group than in the control group, and the median values of log2(*TPM*) are all concentrated around 4. The moderately expressed genes account for more of the total, and the gene expression of the treated group is higher than that of the control group.

### 3.4. Differential Gene Analysis

**Figure 2:**
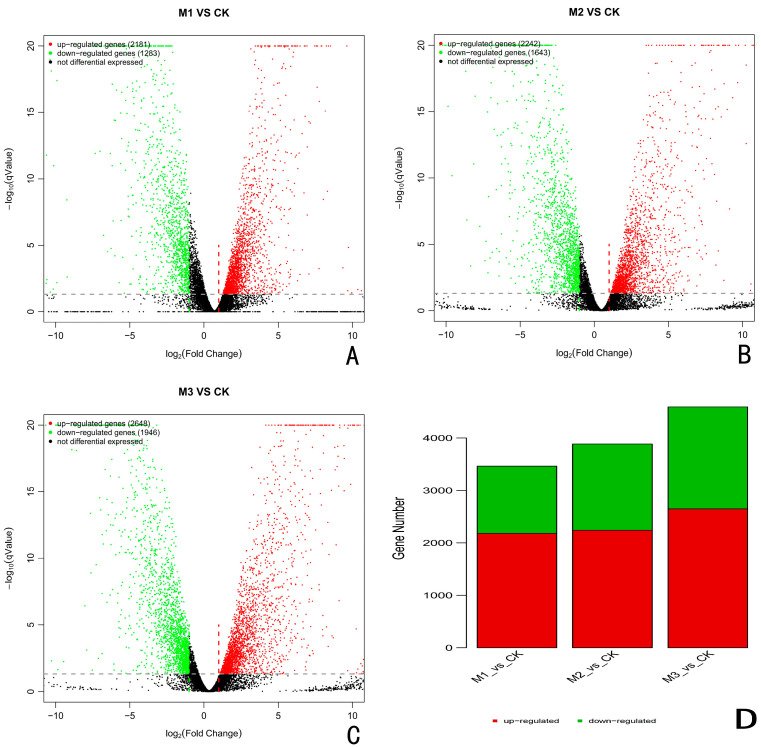
Results of differential gene analysis. (**A**) CK vs. 24 h, (**B**) CK vs. 48 h, (**C**) CK. vs. 72 h. M stands for group, M1 stands for 24 h, M2 stands for 48 h, and M3 stands for 72 h. The horizontal axis is the average log(TPM) of the two groups of samples, that is, (log(A) + log(B))/2, and the vertical axis is log(fold change), that is, the log(B/A) value. Each dot represents a gene, Green dots to the left of the green dashed line and above the gray dashed line indicate down-regulated genes, and red dots to the right of the red dashed line and above the gray dashed line indicate up-regulated genes, and black representing the non-differential genes. (**D**) Statistical map of the differentially expressed genes. The horizontal axis is the difference comparison name, and the vertical axis is the number of down-regulated differential genes. Where the green is down, the red is up.

The differentially expressed genes (DEGs) in *A. fatua* L. infected with *T. polysporum* HZ-31 were screened by the FDR-corrected *p*-value ≤ 0.05 and the absolute value of the log_2_ ratio > 2. The sequencing results for each experimental group and for the control group were analyzed. When compared with the control group, there were 3464 differentially expressed genes, including 1283 down-regulated genes and 2181 up-regulated genes after 24 h of infection by the pathogen (Figure 2A,D). After 48 h of infection, there were 3885 differentially expressed genes, of which 2242 were up-regulated and 1643 were down-regulated (Figure 2B,D). The number of differentially expressed genes after 72 h of infestation was the highest among all the groups, with 4594 differentially expressed genes, of which 2648 were up-regulated and 1946 were down-regulated (Figure 2C,D).

### 3.5. Venn Analysis

**Figure 3:**
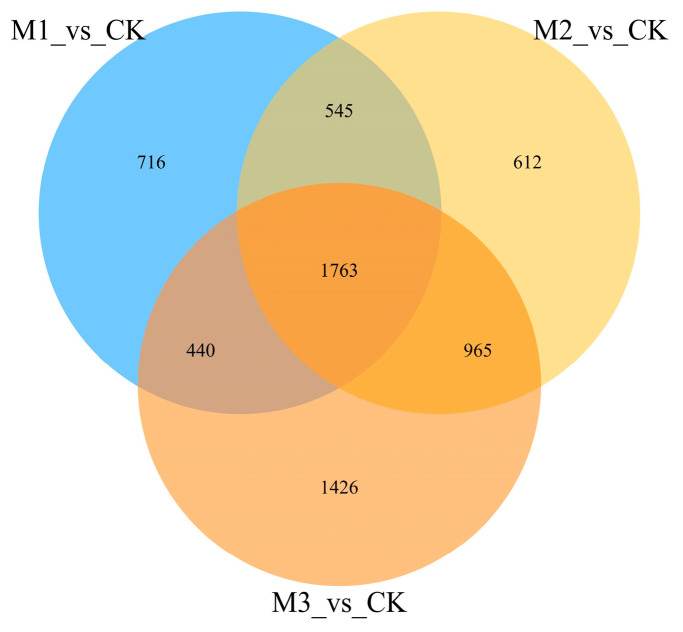
Results of Venn analysis. M stands for group, M1 stands for 24 h, M2 stands for 48 h, and M3 stands for 72 h. Different comparison groups are represented by different colors, and the numbers in the figure represent the number of specific or common differentially expressed genes. The overlapping region represents the number of differentially expressed genes shared by the different comparison groups, while the non-overlapping region represents the number of differentially expressed genes unique to the different comparison groups.

A Venn analysis of the DEGs after the three pathogen-infection time points and comparative analysis showed that 980 genes were co-occurring DEGs in all three groups at all time points, which accounted for a smaller portion of the total. In total, 3960 differentially expressed genes were present (Figure 3).

### 3.6. Enrichment Analysis of Up-Regulated Differentially Expressed Genes

#### 3.6.1. GO Functional Enrichment Analysis of Up-Regulated Differentially Expressed Genes

**Figure 4:**
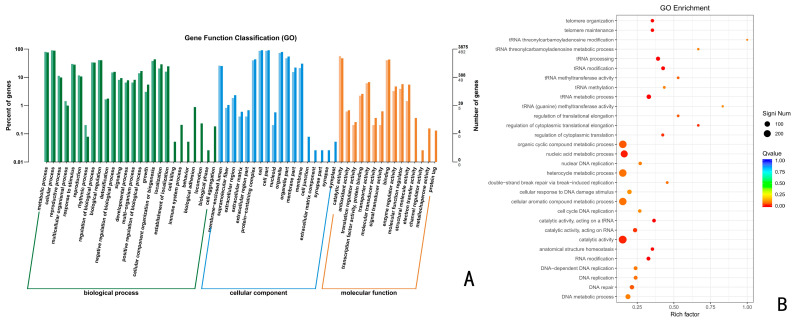
GO functional enrichment analysis of *Trichoderma polysporum* HZ-31 differential genes after treatment with CK vs 24 h. (**A**) The horizontal axis of the differential gene GO annotation classification bar is the functional classification, and different colors represent different classifications. The values on the bar chart and the vertical axis are lighter colored for the differential genes and darker colored for all the genes. The right vertical axis is the number of genes in the classification, and the left vertical axis is the number of genes annotated to the function (differential genes/all genes). (**B**) GO enrichment bubble charts. The vertical axis represents the functional annotation information, and the horizontal axis represents the Rich factor corresponding to the function (the number of differential genes annotated to the function divided by the number of genes annotated to the function). The size of the Q value is represented by the color of the dot. The smaller the Q value is, the closer the color is to red. The number of differential genes contained in each function is expressed by the size of the dots. (We only selected the top 30 GOs with the highest degree of enrichment.) Figure 4, Figure 5 and Figure 6 are the same.

Using the GO database, the above up-regulated DEGs of the CK vs. 24 h, CK vs. 48 h, and CK vs. 72 h were analyzed by GO functional enrichment. The results indicated that the up-regulated DEGs at 24 h, 48 h, and 72 h were distributed among 43, 48, and 45 categories of the three major functional annotations, respectively. A total of 1320 up-regulated differentially differentiated genes were annotated for CK vs. 24 h (Figure 4), with 476 biological processes (BPs), 410 molecular functions (MFs), and 434 cellular components (CCs), accounting for 36.06%, 31.06%, and 32.88%, respectively. These up-regulated differential genes were significantly enriched in the organic cyclic compound metabolic process, nucleic acid metabolic process, heterocycle metabolic process, cellular aromatic compound metabolic process, and catalytic activity, with the highest number of genes.

**Figure 5:**
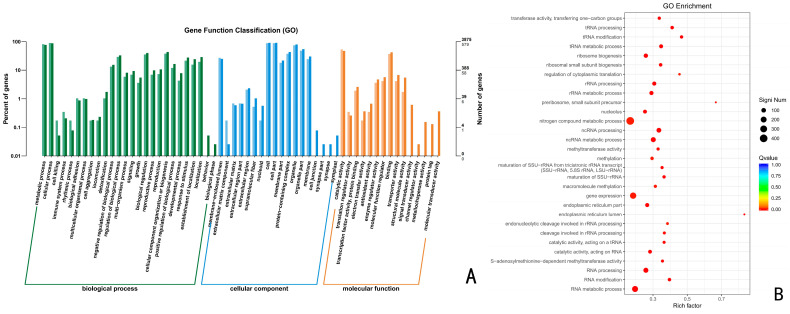
(**A**) GO functional enrichment analysis of *Trichoderma polysporum* HZ-31 differential genes after treatment with CK vs. 48 h. (**B**) GO enrichment bubble charts.

A total of 1542 up-regulated differential genes, including 558 biological processes, 463 molecular functions, and 521 cellular components were annotated for CK vs. 48 h (Figure 5), accounting for 36.19%, 30.03% and 33.79%, respectively. These up-regulated differential genes were significantly enriched in the nitrogen compound metabolic process, gene expression, and RNA metabolic process, with the highest number of genes.

**Figure 6:**
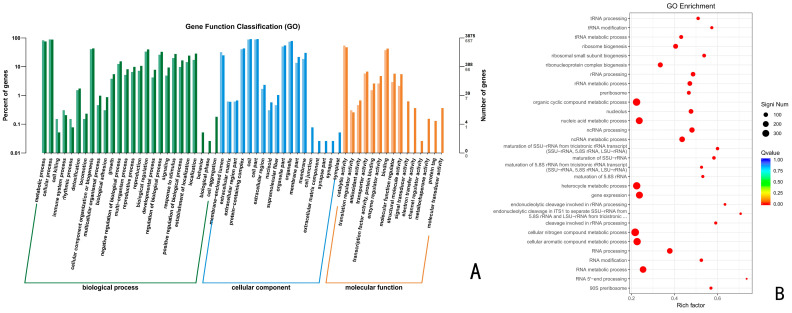
(**A**) GO functional enrichment analysis of *Trichoderma polysporum* HZ-31 differential genes after treatment with CK vs. 72 h. (**B**) GO enrichment bubble charts.

A total of 1767 up-regulated differential genes, including 635 biological processes, 545 molecular functions, and 587 cellular components were annotated by CK vs. 72 h (Figure 6), which account for 35.94%, 30.84%, and 33.22%, respectively. These up-regulated differential genes were significantly enriched in the organic cyclic compound metabolic process, nucleic acid metabolic process, heterocycle metabolic process, gene expression, cellular nitrogen compound metabolic process, cellular aromatic compound metabolic process, and RNA metabolic process, with the highest number of genes.

#### 3.6.2. KEGG Functional Enrichment Analysis of Up-Regulated Differentially Expressed Genes

**Figure 7:**
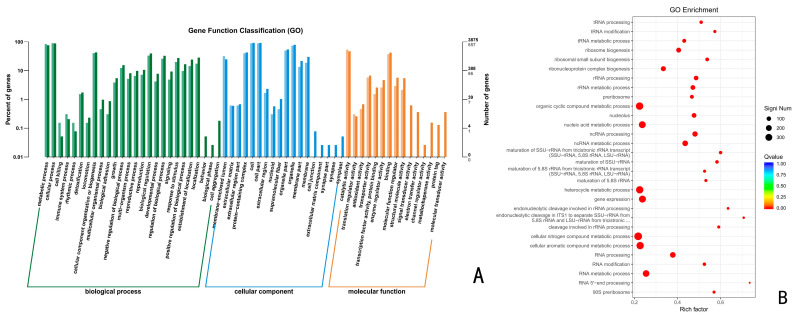
KEGG functional enrichment analysis of *Trichoderma polysporum* HZ-31 differential genes under the different treatments. (**A**) CK vs. 24 h, (**B**) CK vs. 48 h, and (**C**) CK vs. 72 h. The vertical axis represents the functional annotation information, and the horizontal axis represents the Rich factor corresponding to the function (the number of differential genes annotated to the function divided by the number of genes annotated to the function). The size of the Q value is represented by the color of the dot. The smaller the Q value is, the closer the color is to red. The number of differential genes contained in each function is expressed by the size of the dots. (We only selected the top 30 KEGGs with the highest degree of enrichment).

The top 30 KEGG pathways that were significantly enriched in the up-regulated expressed genes compared to the CK at the three different time periods were analyzed after the infection of *A. fatua* L. with *T. polysporum* HZ-31 for 24 h, 48 h, and 72 h (Figure 7). The results show that phenylalanine, tyrosine, and tryptophan biosynthesis; naphthalene degradation; methane metabolism; dioxin degradation; the degradation of aromatic compounds; chlorocyclohexane and chlorobenzene degradation; and the carbapenem biosynthesis pathways appeared in all three groups, suggesting that the above pathways play an important role in the synthesis of herbicidal secondary metabolites by *T. polysporum* HZ-31. Among these pathways, phenylalanine, tyrosine, and tryptophan biosynthesis; the degradation of aromatic compounds; and methane metabolism were associated with the herbicidally active compounds of *T. polysporum* HZ-31, such as P-hydroxyphenyl-2,3-dihydroxypropyl ether, O-hydroxy-3-carbonyl-1-O-hydroxy-3-carbonyl-1-hydroxyphenylpropanol, 1,8-propanediol-o-xylene, and the biosynthesis of P-hydroxyphenyl-2,3-dihydroxypropyl ether.

#### 3.6.3. Metabolic Pathway Analysis

**Figure 8:**
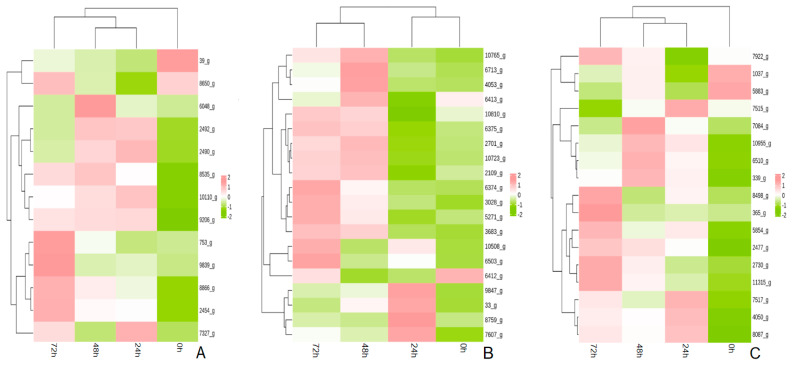
Analysis of the key metabolic pathways of *T. polysporum* HZ-31 under different treatments: (**A**) phenylalanine biosynthesis, (**B**) aromatic compound metabolism, and (**C**) methane metabolism. Each row represents a gene, each column represents a sample, and the color indicates the size of the gene’s expression in the sample, with red representing a higher expression of the gene in the sample and green representing a lower expression.

The phenylalanine biosynthesis pathway was significantly enriched in all three periods after the infection of *A. fatua* L. with *T. polysporum* HZ-31. The phenylalanine biosynthesis pathway was annotated to a multitude of genes in the different periods after the infection of *A. fatua* L. with *T. polysporum* HZ-31 (Figure 8A); the genes that were significantly up-regulated were *TRP* (8866_g), *PHA2* (2454_g), *AATR1* (6048_g), *DHQA* (2492_g), *AATR1* (8535_g), *3DHQ* (2490_g), *AATC* (7327_g), *TRPE* (10110_g), *TRPG* (9206_g), *AROF* (753_g), and *AROM* (9839_g), encoding, respectively, tryptophan synthase, putative prephenate dehydratase, aromatic amino acid aminotransferase, quinate dehydrogenase, aromatic amino acid aminotransferase, catabolic 3-dehydroquinase, aspartate aminotransferase, anthranilate synthase component 1, anthranilate synthase component 2, 3-deoxyheptanose-7-phosphate synthetase and pentafunctional *AROM* polypeptide.

The metabolic pathways of the aromatic compounds were also significantly enriched in all three periods after the infection of *A. fatua* L. by *T. polysporum* HZ-31 (Figure 8B). The significantly up-regulated genes were *CP53* (3028_g), *YJU6* (6374_g), *NHG2* (2109_g), *GNL* (6713_g), *NHG1* (6375_g), *ADH2* (5271_g), and *3HBHI* (10810_g), encoding, respectively, benzoate 4-monooxygenase, the putative transcriptional regulatory protein, salicylate hydroxylase, gluconolactonase, salicylate hydroxylase, and alcohol dehydrogenase 2,3-hydroxybenzoate 6-hydroxylase.

In the methane metabolic pathway (Figure 8C), the genes that were significantly up-regulated during the three periods were *FDH* (5854_g), *DAS* (10855_g), *GDH* (7084_g), *DAS* (6510_g), *ALF* (11315_g), *ALOX* (8087_g), and *PGDH* (4050_g), encoding formate dehydrogenase, dihydroxyacetone synthase, glycerate dehydrogenase, dihydroxyacetone synthase, glycerate dehydrogenase, dihydroxyacetone synthase, and glycerate dehydrogenase, respectively.

### 3.7. Validation of Transcriptome Results

**Figure 9:**
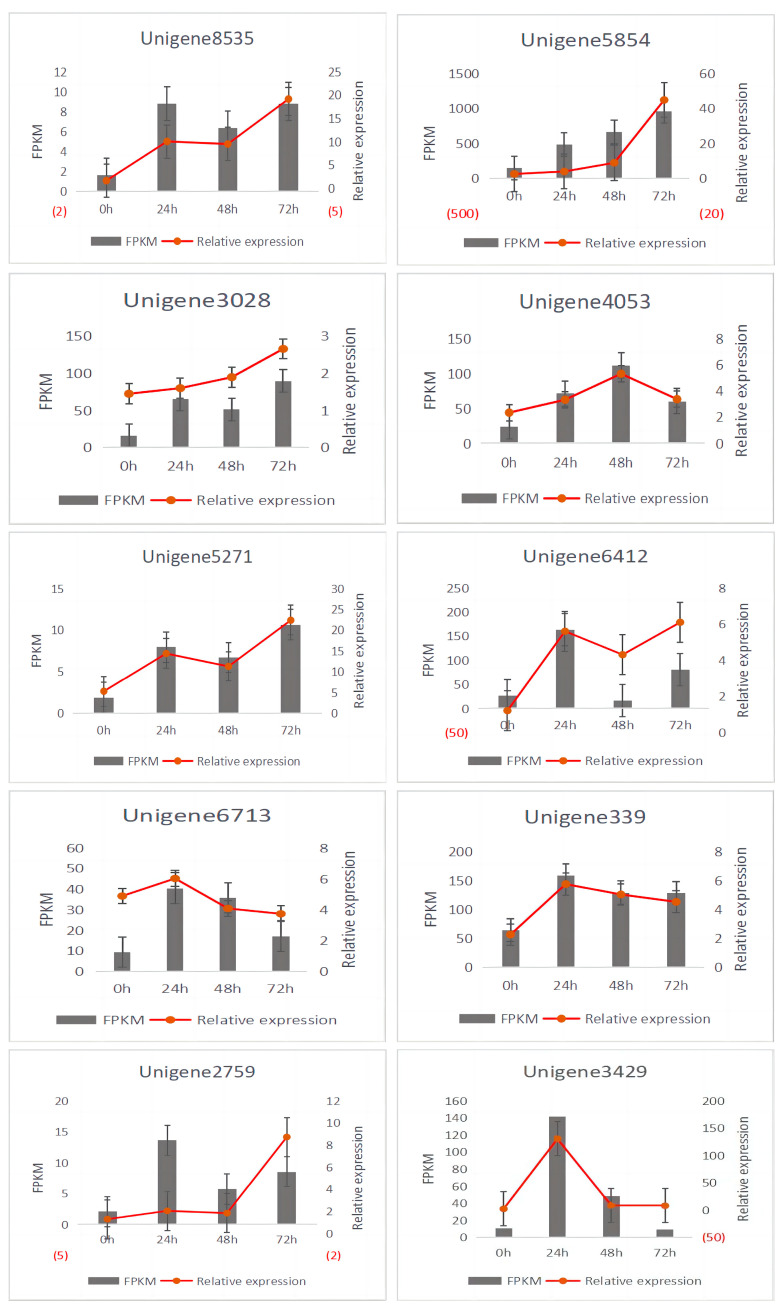

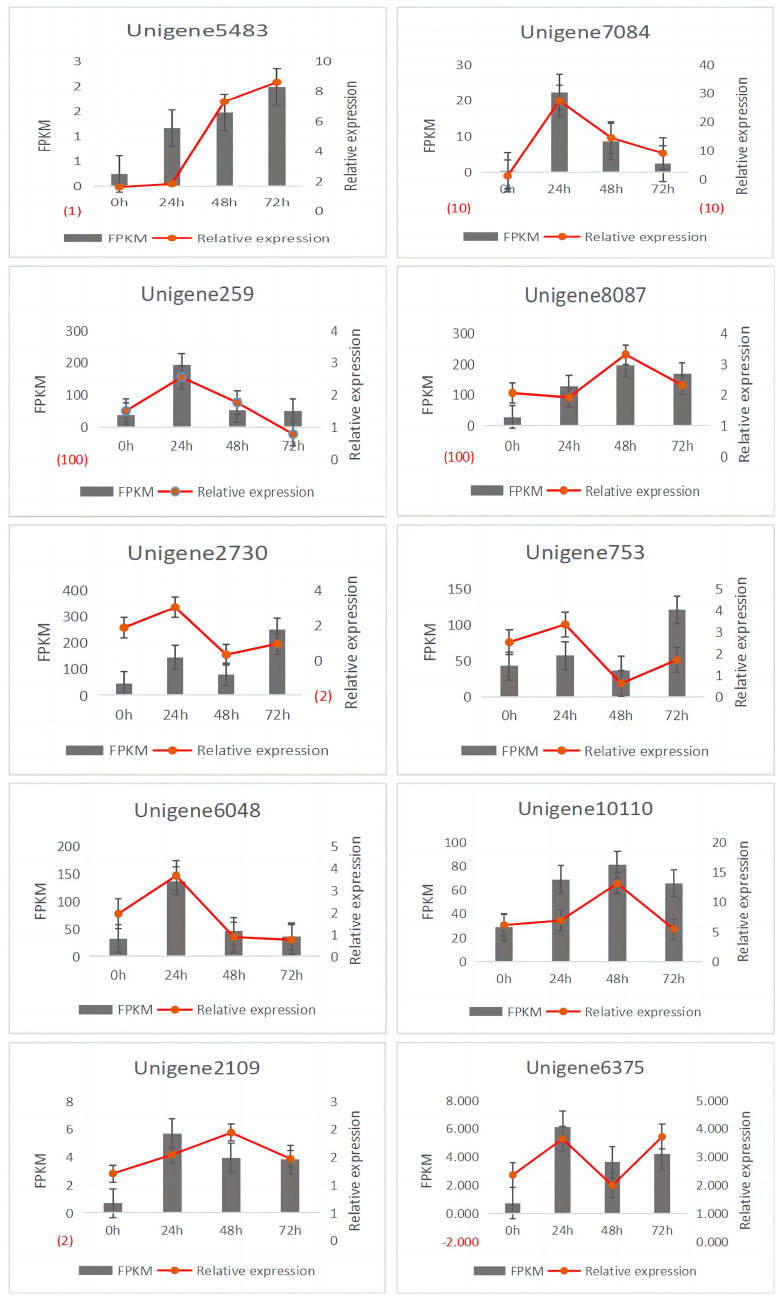
qRT-PCR of the differential genes. The title of the chart represents the i.d. of the different differential genes. The left ordinate represents the FPKM values of the differential genes in the transcriptome. The right ordinate represents the relative expression of the differential genes in the qRT-PCR.

In this experiment, the differential genes were analyzed by qRT-PCR. The FPKM values of the transcriptome sequencing and the relative expressions of the genes that were determined by real-time fluorescence quantification were plotted and analyzed on the vertical axis, and the horizontal axis indicated the different treatment time points. The results are shown in Figure 8. The RNA-seq results for 20 genes were basically consistent with the expression trends of the qRT-PCR sequencing results (Figure 9), which prove that the transcriptome sequencing results are reliable.

## 4. Discussion

The ultimate goal of this study was to understand the differential expression of pathogenic fungal genes and their involvement in metabolic pathways during the infestation of *A. feua* L. leaves by *T. polysporum* HZ-31 by transcriptome sequencing, and to explore the differentially expressed genes related to the pathogenicity of the pathogenic fungi. The results show that there were 3464, 3885, and 4594 differential genes during the three different time periods when *T. polysporum* HZ-31 infected *A. feua* L., showing an increasing trend and an increasing number of up-regulated genes. This is consistent with the results of Wang et al.’s study, which showed that *Trichoderma harzianum* increased the number of up-regulated differential genes over time during parasitism [17]. The trend of the up-regulated differential genes indicates that the infectious activity of *T. polysporum* HZ-31 on *A. feua* L. may increase with time within 72 h after infection. After the up-regulated genes were enriched with the GO function, the number of differential genes enriched that fell into the category of biological processes was more than that for the number of genes in the categories of cell components and molecular functions, indicating that *T. polysporum* has a higher activity of genes related to biological processes during infection, including the GO classification of metabolic processes, cellular processes, biological regulation, etc. The results of the GO enrichment analysis were similar to the transcriptome analysis of *Fusarium solani*-infected sweet potato studied by Luo et al. [18].

To date, at least 545 fungal phytotoxic secondary metabolites have been reported, including 207 polyketides, 46 phenols and phenolic acids, 135 terpenoids, 146 nitrogenous metabolites, and 11 other metabolites. Among them, aromatic polyketides and sesquiterpenoids are the major phytotoxic compounds [19]. The metabolism of phenylalanine, tyrosine, and tryptophan (also known as the three aromatic amino acids) are involved in fungal growth and are known precursors of several mycotoxins. These amino acids can be synthesized and utilized by fungi to produce potentially damaging mycotoxins. In this study, *A. feua* L. was infected with *T. polysporum* HZ-31. During the three periods, the phenylalanine biosynthesis pathway was significantly enriched, indicating that phenylalanine plays an important role in the pathogenicity of *T. polysporum* HZ-31, and it may be a precursor to the synthesis of *T. polysporum* HZ-31 herbicidal active substances. This is consistent with the results of Santiago et al.’s study, which showed that the metabolic process of *Ganoderma boninense* is mainly enriched in the biosynthesis pathway of phenylalanine, tyrosine and tryptophan, and that this pathway plays an important role in the growth and development of *Ganoderma boninense* and toxin synthesis [20]. The theoretical pathway for the microbial production of phenylalanine from glucose involves glucose first generating phosphoenolpyruvic acid and erythrose 4-phosphate via the glycolysis pathway and the pentose phosphate pathway, respectively, which then enters the mangiferic acid pathway to generate mangiferic acid, and phenylalanine is finally generated from mangiferic acid [21]. Among the enzymes, 3-deoxyheptanose-7-phosphate synthetase encoded by *AROF,* and five functional fusion proteins encoded by *AROM* are the first key enzymes of the mangiferic acid synthesis pathway. Their up-regulated expression promotes the biosynthesis of chorismic acid, and the putative *PHA2* gene encoding prephenate dehydratase catalyzes the production of prephenic acid from chorismic acid, which in turn promotes phenol biosynthesis [22]. The proteins encoded by the *AROF*, *AROM*, and *PHA2* genes showed up-regulation in this study, and the expression of the *AROF* and *PHA2* genes in the phenylalanine biosynthesis pathway in the treatment group showed an up-regulated trend compared with the control group, so it is speculated that the *AROF* and *PHA2* genes are key genes in the synthesis of the herbicidal active substances of *T. polysporum* HZ-31.

The metabolic pathway of aromatic compounds in this study was also significantly enriched in the three time periods of *A. feua* L. infestation by *T. polysporum* HZ-31, which is consistent with the result that the KEGG pathway, which was predominantly enriched to be the metabolic pathway of aromatic compounds at the 6th d of apple infestation by the *Valsa mali var.* mali, as shown by Jin et al. [23]. The gene *ADH2,* encoding alcohol dehydrogenase ADHS, is enriched in the metabolic pathway of the aromatic compounds, and the expression level is always up-regulated compared with the control group. Alcohol dehydrogenase ADHS is widely found in plants, animals, microorganisms, and other organisms [24]. In recent years, ADHS has been found to play an important role in the self-growth of many pathogens and their pathogenicity to plants. Smidt [25] found that the transcription levels of the *BcADH1* gene were significantly up-regulated during the tomato–Botrytis cinerea strain B0510 interaction and in the early stages of infection. The virulence of the mutant on tomato leaves after knockout of the gene was significantly different from that of the wild type. These results provide strong evidence for the importance of *ADH1* genes for fungal development, including their environmental adaptation and reaching full pathogenicity [26]. Therefore, it is speculated that the *ADH2* gene encoding alcohol dehydrogenase in this study is involved in the prevention of *A. feua* L. infection by *T. polysporum* HZ-31. The reason for its continued up-regulation during the three periods of *T. polysporum* HZ-31 infection is that it may regulate the growth and development and pathogenicity of *T. polysporum* HZ-31.

Previous studies in our laboratory have shown that the *GDH* gene in the methane metabolic pathway is located in the secondary metabolite synthesis gene cluster encoding enniatin. Structurally, enniatin is a cyclohexadecapeptide composed of alternating residues of three N-methylamino acids (usually valine, leucine, and isoleucine) and three hydroxyl acids (usually hydroxyisovaleric acid). It is a well-known antibacterial, repellent, antifungal, herbicidal, and insecticidal compound. [27]. In order to determine whether the strain virulence was affected by fusarium enniatin, potato tuber tissues were measured. The results showed that 7 strains that produced enniatin and 16 strains that did not produce enniatin induced tissue necrosis of potato tubers. As a result, the production of enniatin by the strains that synthesize it may influence its pathogenicity [28]. In this study, the expression of the *GDH* gene was up-regulated during the infection of *A. feua* L. by *T. polysporum* HZ-31, and the expression level was higher after 24 h of infection. Therefore, it is speculated that the *GDH* gene might be involved in the synthesis of the secondary metabolite of *T. polysporum* HZ-31, thus regulating its pathogenicity.

In this study, we first analyzed the gene expression characteristics of *T. polysporum* HZ-31 during three different periods after infection of *A. fatua* L. at the transcriptome level, and found the key disease-causing genes to be *AROF*, *ADH2*, *PHA2*, and *GDH*, which are related to the synthesis of the secondary metabolites of the pathogen. The biological functions of these genes will be further investigated in the future in order to utilize the herbicidal potential of *T. polymorpha* HZ-31 to develop new and efficient bioherbicides that are suitable for the control of *A. fatua* L. in the Tibetan Plateau region.

## Figures and Tables

**Table 1:** Candidate gene primer sequences.

Gene ID	Forward Primer (5′→3′)	Reverse Primer (5′→3′)	Size (bp)
actin	CCTCCTCTTCCTTGCCAGCATTG	CCTCCTCTTCCTTGCCAGCATTG	111
Unigene2109	TGGCGGTGGTATTGGAGGTCTG	TCCTTGTATGCTGGTGCTTGTTCG	102
Unigene6375	ATTCTGAACGACCGTGAACTCTTGG	TGGTGTGGAAGCATCGCATGTG	96
Unigene8535	GCTGACTGGCAATGCTCTATGAC	CAATGGGCTGGGCGGTCTC	139
Unigene5854	CAATGGGCTGGGCGGTCTC	CAGCGTTCGGAGTGTTGATGG	139
Unigene3028	TCTCGCTCGCTTGATGGAAGG	ATGAGGAGTTGGAGGTCGTATCG	119
Unigene4053	CTTGGCTTAGAATCCCTCGTTGAC	TGATGGTGCCGCCGTTCTC	136
Unigene5271	GATGGCTTCTCCTCTGCTATGTG	TCTGAACTCCAATATGTCCCAAACC	134
Unigene6412	CGAAGATGCTGCTGCGTTAGG	CGAACAGTATCATACACGGCTAGG	90
Unigene6713	TGTTGCGAATGAGCGAGATGC	CCCTTGTAGTTTGTTCCTCCGTTAG	99
Unigene339	GGTTCTCTCTGGCGGTTTCTTG	CCTCGTTAGCATGTGCGTAGTC	80
Unigene2759	TGGCGGACTTGGTGGATGAG	TGCGTGATATTGATAGAGGCTTGC	84
Unigene3429	TGCGAACTGGACTGGACTCTG	GTTGATCTCGGTGACGGCTTTC	110
Unigene5483	TCGCCTATGCCGTGGTTCC	GGTTCGTCCATTCGCCAGATG	150
Unigene7084	GGACCATGAGACCTGATAGCATTG	AGTCGCTGGCTCGGTTACG	137
Unigene259	CAATGCCGCCGATTCACAGG	GGACCTTGTAATAGTTGCCGTATGG	92
Unigene8087	AGGTGTTGGCGAGGAGTATCAG	TCAAGCGTCTCTTCAGTCTTTAGTG	81
Unigene2730	GCCATTGAATGCCTTGCTTGAC	ATGTCCGCCGCCTTGGTAG	102
Unigene753	TCGCTCGGTGTCGCCATC	AAGCAGTCTTCGTTACCTGTTGTG	116
Unigene6048	TCGCCTCGGAGCAGATTGTC	GTTCATCAGCCATCGCAGGTAG	143
Unigene10110	GGCGGCGGCATTGTATTCG	GCGGATTGCTGCTGGTCATAG	125

**Table 2:** Sequencing data quality.

Sample	Raw Reads	Clean Reads	Clean Bases	Q20 (%)	Q30 (%)	GC Content (%)
CK-1	64,467,532	62,859,772	9,198,358,055	99.00%	96.17%	52.39%
CK-2	61,689,040	60,084,542	8,787,122,185	98.98%	96.16%	52.54%
CK-3	55,680,734	54,362,330	7,879,606,421	99.07%	96.47%	53.05%
T24-1	56,045,126	54,310,362	7,791,837,914	98.85%	95.69%	52.06%
T24-2	55,269,126	54,006,616	7,771,363,231	99.03%	96.29%	52.61%
T24-3	61,983,022	60,503,534	8,682,351,056	99.04%	96.32%	52.66%
T48-1	57,216,160	55,900,958	8,163,997,746	99.03%	96.31%	51.87%
T48-2	49,386,632	48,061,810	7,007,420,562	98.97%	96.09%	51.68%
T48-3	51,854,456	50,528,528	7,354,822,322	99.01%	96.19%	51.68%
T72-1	77,526,690	75,760,238	10,964,415,778	99.06%	96.41%	52.34%
T72-2	59,115,428	57,610,338	8,362,733,753	99.01%	96.24%	52.51%
T72-3	58,408,616	56,724,148	8,171,046,956	98.93%	95.97%	51.80%

Note: CK is the control group and T is the treatment group. Sample: indicates the sample name. Clean reads: data after QC. Q20 means that the sequencing error rate of this base is 0.01, and Q30 means that the sequencing error rate of this base is 0.001; therefore, the higher the ratio of Q20 and Q30, the better the data quality.

**Table 3:** Reads in the reference genome alignment results.

Sample	Clean Reads	Total Mapped	Multiple Mapped	Uniquely Mapped	Read1 Mapped	Read2 Mapped	Proper Mapped
CK-1	56,568,506 (100.00%)	56,110,923 (99.19%)	163,743 (0.29%)	55,947,180 (98.90%)	27,979,165 (49.46%)	27,968,015 (49.44%)	54,648,680 (96.61%)
CK-2	59,334,990 (100.00%)	58,694,852 (98.92%)	238,005 (0.40%)	58,456,847 (98.52%)	29,220,950 (49.25%)	29,235,897 (49.27%)	57,088,794 (96.21%)
CK-3	54,102,082 (100.00%)	53,611,134 (99.09%)	176,271 (0.33%)	53,434,863 (98.77%)	26,719,566 (49.39%)	26,715,297 (49.38%)	51,866,660 (95.87%)
T24-1	50,455,296 (100.00%)	49,908,731 (98.92%)	147,119 (0.29%)	49,761,612 (98.63%)	24,899,621 (49.35%)	24,861,991 (49.28%)	48476952 (96.08%)
T24-2	53,627,174 (100.00%)	52,963,908 (98.76%)	179,316 (0.33%)	52,784,592 (98.43%)	26,400,523 (49.23%)	26,384,069 (49.20%)	50,871,496 (94.86%)
T24-3	59,771,456 (100.00%)	59,042,883 (98.78%)	209,930 (0.35%)	58,832,953 (98.43%)	29,428,530 (49.24%)	29,404,423 (49.19%)	56,860,444 (95.13%)
T48-1	52,454,282 (100.00%)	51,511,562 (98.20%)	238,042 (0.45%)	51,273,520 (97.75%)	25,640,147 (48.88%)	25,633,373 (48.87%)	49,681,412 (94.71%)
T48-2	34,189,154 (100.00%)	33,535,178 (98.09%)	114,753 (0.34%)	33,420,425 (97.75%)	16,714,982 (48.89%)	16,705,443 (48.86%)	32,285,094 (94.43%)
T48-3	29,116,484 (100.00%)	27,919,427 (95.89%)	94,113 (0.32%)	27,825,314 (95.57%)	13,918,967 (47.80%)	13,906,347 (47.76%)	26,793,860 (92.02%)
T72-1	75,273,746 (100.00%)	74,106,054 (98.45%)	345,027 (0.46%)	73,761,027 (97.99%)	36,888,884 (49.01%)	36,872,143 (48.98%)	71,239,844 (94.64%)
T72-2	57,056,046 (100.00%)	56,079,845 (98.29%)	281,043 (0.49%)	55,798,802 (97.80%)	27,908,963 (48.91%)	27,889,839 (48.88%)	53,954,714 (94.56%)
T72-3	54,409,280 (100.00%)	53,175,243 (97.73%)	320,973 (0.59%)	52,854,270 (97.14%)	26,441,123 (48.60%)	26,413,147 (48.55%)	51,321,778 (94.33%)

## Data Availability

The data that support the findings of this study are available from the corresponding author upon reasonable request.
